# Isolation and Characterisation of Acid Soluble Collagens and Pepsin Soluble Collagens from Eel (*Anguilla japonica* Temminck et Schlegel) Skin and Bone

**DOI:** 10.3390/foods14030502

**Published:** 2025-02-05

**Authors:** Ningning Zhang, Shaoli Guo, Yuting Zheng, Weili Li

**Affiliations:** 1 Functional Food Research Group, Division of Allied Health, School of Allied and Public Health, University of Chester, Chester CH1 4BJ, UK; n.zhang@chester.ac.uk; 2 Engineering Research Centre of Fujian-Taiwan Special Marine Food Processing and Nutrition (Ministry of Education), Fujian Agriculture and Forestry University, Fuzhou 350002, China; gslfafu@163.com (S.G.); zyteen@163.com (Y.Z.)

**Keywords:** eel by-products, collagen, structural characterisation, thermal stability, amino acid composition

## Abstract

Eel (*Anguilla japonica*) is an important and valuable food fish in East Asia and its by-products have been reported to include bioactive and profitable components. This study aimed to extract, characterise, and compare the structure and properties of acid-soluble collagens (ASCs) and pepsin-soluble collagens (PSCs) from the skin and bone of eel (*Anguilla japonica*), providing insights into their composition, structure, and properties for various applications. The yields of ASC-S (from skin), PSC-S (from skin), ASC-B (from bone), and PSC-B (from bone) were 12.16%, 15.54%, 0.79%, and 1.34% on a dry weight basis, respectively. Glycine, the dominant amino acid, accounted for 16.66% to 22.67% of total amino acids in all samples. SDS-PAGE and FTIR analyses showed the typical triple-helical structure of type I collagen with slight variations in molecular order in extract and intermolecular cross-linking between skin and bone collagens. The denaturation temperature (*T_max1_*) measured by differential scanning calorimetry (DSC) is 81.39 °C and 74.34 °C, respectively, for ASC-B and ASC-S. Bone collagen has higher thermal resistance than skin collagen. Surface morphology imaged using a scanning electron microscope (SEM) showed that the bone collagen had a denser network structure, whilst the skin collagen was more fibrous and porous. The findings suggest that eel-derived collagens from skin and bone can serve as potential alternatives in the food, cosmetic, and healthcare industries.

## 1. Introduction

Collagen, a pivotal biological macromolecule, constitutes the main structural component of animal connective tissues and accounts for 25–30% of the total protein in the body. Among its 29 known types, Type I collagen is the most abundant, predominantly found in connective tissues such as skin, bones, and tendons. The unique triple-helical structure of collagen enables its diverse bioactive functions, including antioxidant, angiotensin-converting enzyme (ACE) inhibitory, and immunomodulatory activities, making it a critical material in the food, cosmetics, biomedical, and pharmaceutical industries [1,2,3]. In recent years, collagen sourced from land mammals such as cows and pigs has faced challenges due to concerns over disease transmission and cultural restrictions. This has spurred interest in alternative sources, particularly from aquatic species, which offer advantages such as lower antigenicity and hypoallergenicity [4].

Fish by-products such as skin, bones, and scales are particularly rich in collagen and are increasingly recognised for their sustainability and potential economic value. Numerous studies have successfully extracted collagen from fish, including hybrid sturgeon and tilapia, highlighting its viability as a substitute for mammalian collagen in medical biomaterials and other applications [5,6,7]. However, there remains a lack of comprehensive studies on the structural and functional properties of collagen derived from eel (*Anguilla japonica*), despite its nutritional and medicinal significance in East Asia and its contribution to substantial by-product waste during processing.

This study focuses on extracting and characterising acid-soluble collagen (ASC) and pepsin-soluble collagen (PSC) from the skin and bone of eel. Previous research has identified Type I collagen in eels [8], but comparative insights into the structural and functional properties of collagen from different eel tissues are limited. By employing methods such as SDS-PAGE, FTIR spectroscopy, and SEM imaging, this study aims to elucidate the molecular, thermal, and morphological characteristics of eel-derived collagen. These findings not only expand our understanding of collagen’s diversity across tissues but also reinforce its potential applications in industries ranging from food and cosmetics to healthcare.

## 2. Materials and Methods

### 2.1. Materials and Reagents

Eels (*Anguilla japonica*) were purchased from Yong Hui Supermarket (Fuzhou, China) and stored at −20 °C until use. Pepsin (enzyme activity: 3000 U/mg) was obtained from Sigma Chemical Co. (St. Louis, MO, USA). L-Hydroxyproline was purchased from Beijing Solarbio Science & Technology Co., Ltd. (Beijing, China). All other chemical reagents, including sodium hydroxide, butyl alcohol, acetic acid, and hydrochloric acid, were of analytical grade.

### 2.2. Proximate Composition Analysis

Moisture, crude protein, crude fat, and ash contents were determined using standard AOAC methods [8]. Moisture content was determined by drying the samples in an oven at 105 °C until a constant weight was reached. Crude protein content was measured using the macro-Kjeldahl method, with the nitrogen content converted to protein by applying a factor of 6.25. Crude fat content was assessed by extracting lipids with petroleum ether using a Soxhlet apparatus, followed by evaporation of the solvent and gravimetric quantification of the extracted fat. Ash content was analysed by incinerating the samples in a muffle furnace at 500 °C until the residue was completely ashed.

### 2.3. Preparation of Eel Skin and Bone

Skin and bone were separated from the eels, cut into small pieces, and washed with distilled water. The samples were soaked in 0.1 mol/L sodium hydroxide at a sample-to-solution ratio of 1:10 (m/V) for 48 h to remove non-collagenous proteins, with the solution replaced every 12 h. Afterwards, the samples were rinsed with cold distilled water (4 °C) until a neutral pH was achieved. Lipids were removed by soaking the samples in 10% butyl alcohol for 48 h, with the solution replaced every 12 h. The defatted samples were thoroughly washed with cold distilled water until the wash water was clear. All procedures were conducted at 4 °C to maintain sample integrity [9].

### 2.4. Isolation of Collagen from Eel Skin and Bone

#### 2.4.1. Skin Collagen

Collagen extraction from eel skin was performed with modifications to the method described by Nagai et al. [10] and Senaratne et al. [9]. The pretreated skin was soaked in 0.5 mol/L acetic acid at a sample-to-solution ratio of 1:10 (m/V) for 24 h to extract acid-soluble collagen (ASC). The mixture was filtered through two layers of cheesecloth, and the filtrates were collected. The residue was re-extracted under the same conditions, and the filtrates were combined. Sodium chloride was added to the filtrates to a final concentration of 2.6 mol/L to salt out collagen. The precipitate was collected by centrifugation at 15,000× *g* for 30 min at 4 °C using a refrigerated centrifuge (3H24R1, Hunan Xiangyi Laboratory Instrument Development Co., Ltd., Changsha, China). The precipitate was dissolved in 0.5 mol/L acetic acid, dialyzed sequentially against 0.1 mol/L acetic acid for 48 h and distilled water for another 48 h (solution changed every 12 h), and lyophilised to obtain ASC from skin (ASC-S).

The residue from the ASC-S extraction was further processed to prepare pepsin-soluble collagen (PSC-S). The residue was soaked in 0.5 mol/L acetic acid containing 2% pepsin for 24 h and filtered through cheesecloth. The filtrates were pooled, salted out, and dialyzed as described above. The lyophilised product was PSC-S.

#### 2.4.2. Bone Collagen

The bone collagen was extracted following the method from DeNiro et al. with modifications [11]. The pretreated bones were decalcified by steeping them in 6 mol/L hydrochloric acid for 3 h, with the solution replaced hourly. The samples were washed with cold distilled water until a neutral pH was achieved. ASC from bone (ASC-B) and PSC from bone (PSC-B) were extracted using the same protocols as described for skin collagen.

### 2.5. Measurement of Hydroxyproline Content

The hydroxyproline content was determined using a modified version of the ISO reference method [12]. The samples were mixed with 6 mol/L sulfuric acid and hydrolysed for 6 h at 105 °C. The hydrolysate then was diluted, and 4 mL of diluted hydrolysate was mixed with 2 mL of isopropanol in citrate acetate buffered chloramine T (7 g/100 mL chloramine T solution with acetate-citrate buffer). The mixture was allowed to stand at room temperature for 20 min. Next, 2 mL of the colour-developing agent (100 g of p-dimethylenbenzaldehyde reagent was dissolved in 150 mL perchloric acid solution with 60% mass fraction and then mixed with isopropanol alcohol at a volume ratio of 2:13) was added to the mixture, which then was incubated in a water bath for 20 min at 60 °C. The absorbance of the complex at 558 nm was measured. There was also a hydroxyproline standard solution with concentrations of 0, 0.5, 1, 1.5, and 2 μg/mL.

### 2.6. Collagen Purity and Extractable Yield

The collagen content in the sample was calculated based on the hydroxyproline content, using a collagen conversion factor of 11.1 [13]. The purity of collagen was calculated using the following equation:

(1) $$\%\;Collagen\;purity=\frac{M_{hydroxyprolinecontent}*F}{M_{freeze-dried\;Sample}}*100$$

*M_hydroxyproline content_*—weight of hydroxyproline in freeze-dried acid- or enzyme-extracted samples; F—conversion factor used to calculate collagen content from hydroxyproline is 11.1; *M_freeze-dried Sample_*—weight of freeze-dried acid- or enzyme-extracted samples.

The yield of collagen extractables was calculated using the following equation [14]:

(2) $$\%\;Extractables\;yield\;\left(dry\;basis\right)=\frac{M_{hydroxyprolinecontent}*F}{M_{initial,\;dry}}*100$$

*M_initial, dry_*—weight of dry eel skin or bone

### 2.7. Amino Acid Composition Analysis

The amino acid composition was determined using a modified acid hydrolysis method as described by Ahmed et al., with slight adjustments to the parameters [15]. Samples (200 mg) were hydrolysed with 10 mL of 6 mol/L hydrochloric acid under nitrogen at 110 °C for 24 h. The hydrolysates were centrifuged at 5000× *g* for 30 min at 4 °C, and the supernatant was analysed using an amino acid analyser (L8900, Hitachi High-Tech Corporation, Tokyo, Japan).

### 2.8. SDS-PAGE

SDS-PAGE was performed to analyse collagen patterns. Samples were dissolved in distilled water containing 5% SDS to a concentration of 10 mg/mL and incubated at 85 °C for 1 h. After centrifugation at 10,000× *g* for 10 min, the supernatants were mixed with a loading buffer, boiled, and loaded onto a gel (7.5% separating gel, 5% stacking gel). Gels were stained with 0.2% Coomassie brilliant blue and decolourised by boiling [16].

### 2.9. Fourier Transform Infrared (FTIR) Spectroscopy

FTIR spectra of collagen samples were measured using a Vertex 70 spectrometer (Bruker Co., Ettlingen, Germany). Samples (1 mg) were mixed with 200 mg potassium bromide, pressed into pellets, and scanned over 4000–400 cm⁻^1^ [17].

### 2.10. Scanning Electron Microscopy (SEM)

The microstructure of collagen samples was observed using SEM (Nova Nano SEM 230, FEI, Hillsboro, OR, USA) after sputter coating with gold. Images were captured at magnifications of 500×, 2000×, and 5000× [18].

### 2.11. Differential Scanning Calorimetry (DSC)

The DSC profiles of ASCs and PSCs were analysed using a differential scanning calorimeter (DSC 214, Netzsch Co., Bayreuth, Germany), following the method described by Safandowska et al. [19], with appropriate modifications. Briefly, an accurately weighed amount of lyophilised collagen was placed in a sealed aluminium pan. The samples were scanned and heated from 0 to 200 °C at a heating rate of 10 °C/min, with a nitrogen flow rate of 50 mL/min in the sample chamber. An empty aluminium pan was used as the reference. The maximum denaturation temperature (*T_max_*) was determined as the peak temperature of each endothermic transition, while the total denaturation enthalpy (Δ*H*, J/g protein sample) was calculated from the area under the corresponding endothermic peak.

### 2.12. Ultraviolet (UV) Absorption Spectroscopy

The UV spectrum of the collagen solution was analysed using a UV spectrophotometer (Nicolet iN10, Thermo Fisher Scientific Inc., Waltham, MA, USA). Lyophilised collagen was dissolved in 0.5 mol/L acetic acid to prepare a 0.5 mg/mL solution. Baseline calibration was performed using 0.5 mol/L acetic acid as the blank. UV spectra were recorded in the range of 190–400 nm at 0.5 nm intervals, with a slit width set to 5 nm [20].

### 2.13. Statistical Analysis

Statistical analyses were conducted using SPSS software (version 29.0.1.0). Results were presented as mean ± standard deviation (SD). Differences among groups were evaluated using one-way analysis of variance (ANOVA), followed by Tukey’s post hoc test for multiple comparisons. A *p*-value of < 0.05 was considered statistically significant.

## 3. Results

### 3.1. Approximate Component Analysis

Based on the data presented in Table 1, eel skin and eel bone show distinct differences in their proximate composition. Eel skin has a significantly higher moisture mass fraction of 75.54% compared to eel bone, which contains 46.25%. Both materials are rich in crude protein, with eel skin containing 19.00% and eel bone slightly higher at 20.86%. Fat content varied significantly between eel skin and bone. The fat content of eel bone (including the head) was significantly higher (25.92 ± 0.32%) compared to eel skin (1.40 ± 0.08%). This trend is consistent with findings in other fish species, such as deep-sea redfish, where bone fat content (26.8%) markedly exceeded that of skin (2.9%) [21], and bigeye snapper, where bone fat content (8.77%) was significantly higher than skin fat content (0.98%) [22]. The high fat content in eel bone may be attributed to the presence of bone marrow, which serves as a primary lipid storage site. Additionally, since the eel bone samples used in this study included the skull, the presence of fatty tissues in the head region may have contributed to the observed high fat content.

These findings highlight the unique nutritional profiles of eel skin and bone, which could guide their utilisation in different applications. For instance, the mineral-rich nature of eel bone could be leveraged in mineral-fortified formulations.

### 3.2. Yield Analysis of ASCs and PSCs from Eel Skin and Bone

Collagen extraction from eel skin and bone resulted in yields of 12.16% (ASC-S), 15.54% (PSC-S), 0.79% (ASC-B), and 1.34% (PSC-B), calculated on a dry weight basis. Their purity levels were 85.70% (ASC-S), 80.80% (PSC-S), 83.75% (ASC-B), and 85.16% (PSC-B), respectively. The extraction data suggested that acetic acid alone could not fully extract collagen, particularly from bone. This phenomenon might be attributed to the presence of cross-linking formed by the reaction of aldehydes with lysine and hydroxylysine residues, which reduce the solubility of bone collagen. To improve the yield, pepsin was employed as a supplemental enzymatic treatment. Pepsin, an aspartic protease, specifically breaks peptide bonds at the carboxyl side of phenylalanine, tyrosine, and tryptophan residues. This enzyme enhances collagen solubility by cleaving non-helical telopeptides and facilitating the release of the collagen triple helix. This process makes the collagen more accessible for further extraction.

Interestingly, the yields of PSCs from both skin and bone were higher than those of ASCs, consistent with observations in other species. For example, Wang et al. [23] reported yields of 22.42% for ASC and 27.32% for PSC from loach skin, while Faralizadeh et al. [24] observed yields of 2.24% (ASC) and 9.62% (PSC) from silver carp skin. On the other hand, some studies have shown higher ASC yields, such as Barzkar et al. [25] for fringescale sardinella and Indriani et al. [26] for *Asian bullfrog*. These variations suggest that collagen yield is species-dependent, reflecting differences in biochemical composition and structural properties of the source materials.

### 3.3. Amino Acid Composition of Eel Collagen

Collagen consists of 18 amino acids (Table 2), with glycine (Gly) being the most abundant, followed by proline (Pro) and hydroxyproline (Hyp) in the characteristic Gly-X-Y sequence. In eel bone collagen, glycine accounts for 19.94% (ASC-B) and 16.66% (PSC-B) of the total amino acids, whereas in eel skin collagen, glycine contributes 22.67% (ASC-S) and 19.86% (PSC-S). These proportions align with the typical amino acid composition of collagen, as reported in related studies [15]. Generally, acid-soluble collagens (ASCs) exhibit higher amino acid contents compared to pepsin-soluble collagens (PSCs), with the exception of specific amino acids, including valine (Val), methionine (Met), leucine (Leu), tyrosine (Tyr), arginine (Arg), proline (Pro), and hydroxyproline (Hyp).

All four collagen types contain seven essential amino acids—Met, Leu, Val, lysine (Lys), isoleucine (Ile), threonine (Thr), and phenylalanine (Phe)—which account for 16.32% (ASC-S), 15.40% (PSC-S), 20.58% (ASC-B), and 19.83% (PSC-B) of the total amino acid content. Hydrophobic amino acids make up 30–36% of the total amino acids, including Leu, Val, histidine (His), Tyr, and Pro, which contribute significantly to the antioxidant properties of collagen. Notably, amino acids such as alanine (Ala), Thr, Val, Pro, Ile, Leu, Met, and Phe, located in the C-terminal region, have been linked to angiotensin-converting enzyme (ACE) inhibitory activity [27]. In addition, aromatic amino acids, which constitute approximately 2.3–2.6% of the total amino acid content, further enhance the antioxidant properties of collagen due to their specific side chain structures [28].

These findings suggest that eel-derived collagen is a promising source for the production of bioactive peptides with antioxidant potential, aligning with the growing interest in natural compounds for health and cosmetic applications.

### 3.4. SDS-PAGE Analysis

**Figure 1:**
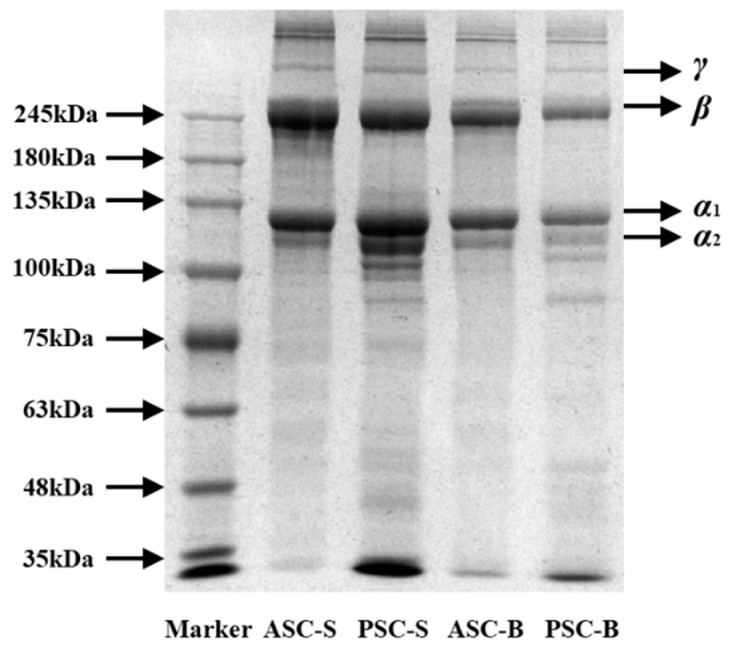
SDS-PAGE of collagen isolated from eel fish skin and bone.

Figure 1 illustrates the SDS-PAGE patterns of collagen extracted from eel skin and bone, highlighting distinct protein profiles for ASC and PSC. The electrophoretic results confirmed the presence of type I collagen, characterised by two different α chains, α1 and α2, and their associated β chains. Notably, the molecular weights of the α1 and α2 chains were approximately 100–135 kDa, while the β chains were around 245 kDa. These findings align with previous studies, such as those involving collagen extracted from Miiuy croaker scales (*Miichthys miiuy*) [29] and channel catfish skin (*Ictalurus punctatus*) [30], which reported similar molecular weight profiles.

The electrophoretic patterns also revealed subtle differences between ASCs and PSCs. The PSC samples displayed more pronounced bands within the molecular weight range of 100 kDa, accompanied by additional smaller fragments below 35 kDa compared to ASCs. This phenomenon is likely attributable to the enzymatic activity of pepsin during PSC extraction, which partially cleaves collagen into smaller peptide fragments while preserving its primary structural components. The greater density of the α1 chains compared to α2 chains across all samples further supports the classification of the extracted collagen as type I.

The SDS-PAGE analysis verified that the collagen extracted from eel skin and bone predominantly comprised type I collagen. The observed differences between ASCs and PSCs underscore the influence of the extraction process on collagen’s molecular integrity.

### 3.5. FTIR Analysis of Collagens Extracted from Eel Skin and Bone

**Figure 2:**
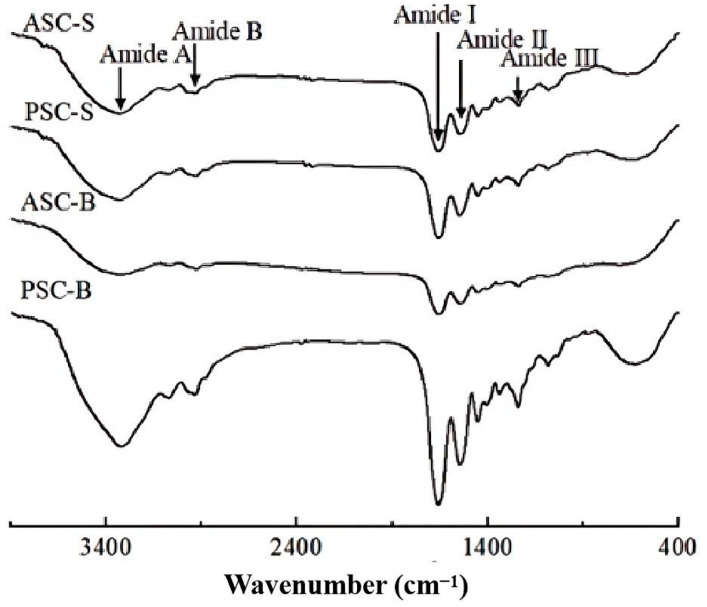
FTIR spectra of collagen isolated from eel fish skin and bone.

Figure 2 presents the FTIR spectra of collagens isolated from the skin and bone of eel. These spectra exhibited characteristic absorption peaks of type I collagen, namely amides A, B, I, II, and III, which provide insights into the molecular structure and secondary conformations of collagen.

The amide A band is related to N–H stretching vibrations, which occur between 3400 and 3440 cm⁻^1^ for free N–H groups. When N–H participates in hydrogen bonding, the stretching frequency decreases [7]. The amide A bands for ASC-S and PSC-S were observed at 3326 cm⁻^1^ and 3327 cm⁻^1^, respectively, whereas those for ASC-B and PSC-B appeared at 3322 cm⁻^1^ and 3326 cm⁻^1^. This shift indicates the involvement of N–H groups in hydrogen bonding, suggesting strong structural stability of the extracted collagen.

The amide B band, which arises from asymmetric CH₂ stretching vibrations, reflects the integrity of the protein’s tertiary structure. The observed amide B peaks were located at 2931 cm⁻^1^ (ASC-S), 2932 cm⁻^1^ (PSC-S), 2924 cm⁻^1^ (ASC-B), and 2933 cm⁻^1^ (PSC-B), consistent with previous findings on collagens extracted from various fish species [31].

The amide I band, appearing between 1600 and 1700 cm⁻^1^, is primarily associated with C=O stretching vibrations coupled with N–H bending and C–N stretching. This band is particularly sensitive to the secondary structure of proteins. The amide I peaks for ASC-S, PSC-S, ASC-B, and PSC-B were observed at 1662 cm⁻^1^, 1661 cm⁻^1^, 1657 cm⁻^1^, and 1660 cm⁻^1^, respectively. Collagens from eel skin exhibited higher wavenumbers, indicative of a greater degree of molecular order compared to those from eel bone. A lower amide I wavenumber is associated with increased hydrogen bonding and a reduction in molecular order [31,32,33]. Previous studies have suggested that β-turns in the amide I region are commonly associated with absorption bands around 1670, 1683, 1688, and 1694 cm⁻^1^, with a characteristic band near 1665 cm⁻^1^. On the other hand, random coils, which are linked to non-repetitive structures, are usually observed in the lower wavenumber region (1640–1648 cm⁻^1^) [34]. In the present study, the observed peak values in the amide I region ranged from 1660 to 1670 cm⁻^1^. Based on these observations, it is suggested that these peaks are primarily attributed to the predominance of β-turn structures, with only minor contributions from α-helices and random coils.

The amide II band, corresponding to N–H bending vibrations and C–N stretching, typically occurs between 1500 and 1600 cm⁻^1^. In this study, the amide II peaks were found at 1545 cm⁻^1^ (ASC-S), 1546 cm⁻^1^ (PSC-S), 1544 cm⁻^1^ (ASC-B), and 1546 cm⁻^1^ (PSC-B). These consistent values suggest that N–H groups in eel collagens were engaged in bonding with adjacent α-chains [32].

The amide III band arises from CH₂ wagging vibrations of glycine backbones and proline side chains, typically appearing in the range of 1200–1300 cm⁻^1^. In this study, the amide III peaks were identified at 1233 cm⁻^1^ (ASC-S), 1236 cm⁻^1^ (PSC-S), 1236 cm⁻^1^ (ASC-B), and 1235 cm⁻^1^ (PSC-B). These results indicate that the triple-helical structure of the collagens was well-preserved across all samples [31].

The FTIR spectra confirmed the typical infrared absorption peaks of type I collagen, consistent with SDS-PAGE analysis. While the structural properties of collagens from eel skin and bone were generally similar, collagen from eel skin exhibited a higher degree of molecular order and intermolecular cross-linking compared to bone collagen. These differences likely result from the distinct structural compositions of skin and bone tissues.

### 3.6. Morphological Analysis Using SEM

The SEM images clearly reveal the fibrous and porous network structures of different collagen samples, reflecting the significant effects of extraction methods and tissue sources on their microstructures.

**Figure 3:**
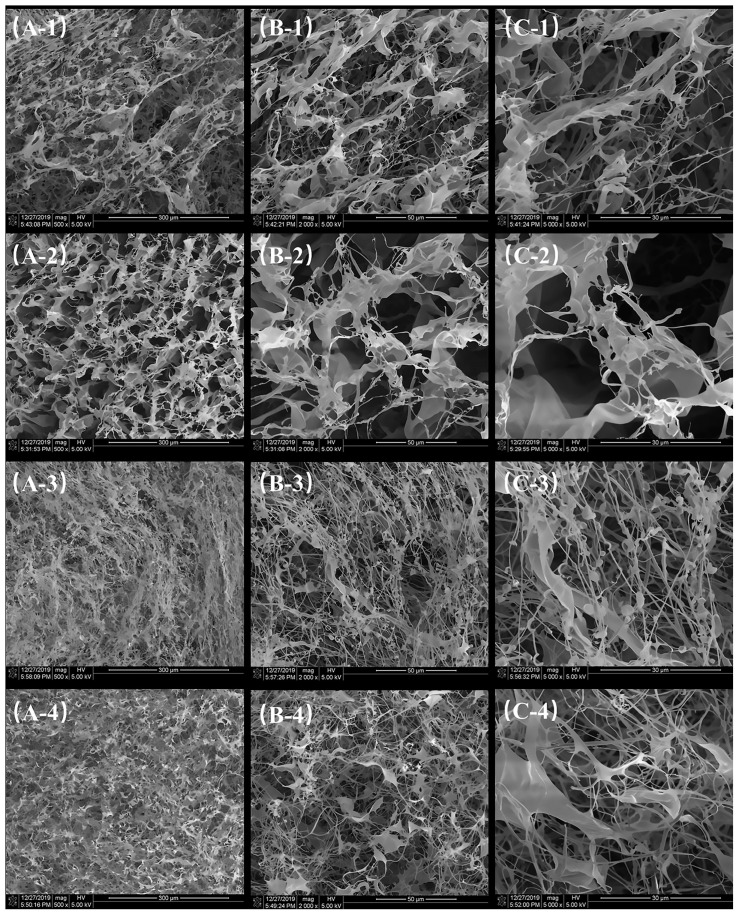
SEM images of (**A-1**,**B-1**,**C-1**) ASC-S, (**A-2**,**B-2**,**C-2**) PSC-S, (**A-3**,**B-3**,**C-3**) ASC-B, and (**A-4**,**B-4**,**C-4**) PSC-B. (**A**), ×500; (**B**), ×2000; (**C**), ×5000.

**Figure 4:**
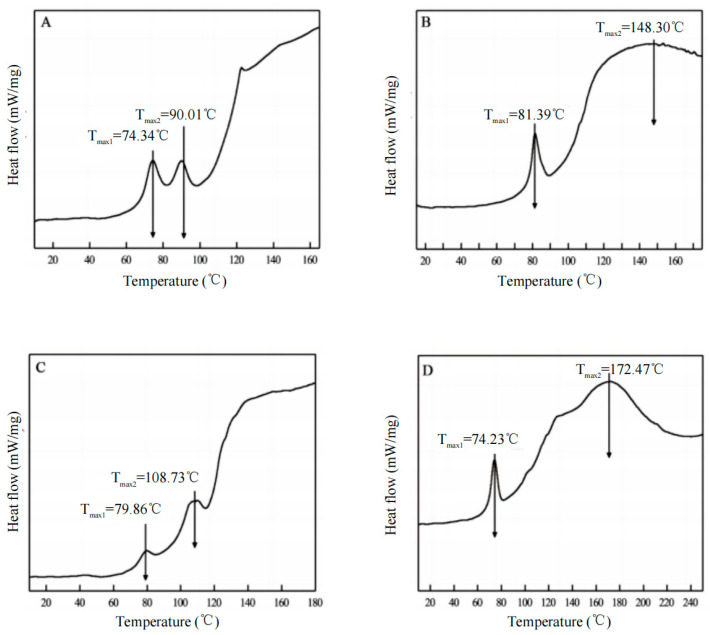
DSC thermograms of collagen isolated from eel fish skin and bone. (**A**), ASC-S; (**B**), ASC-B; (**C**), PSC-S; (**D**), PSC-B.

At lower magnifications (×500; Figure 3A-1,A-2,A-3,A-4), both ASC-S (Figure 3A-1) and PSC-S (Figure 3A-2) exhibit loose fibrous networks with uniform and interconnected pores, without noticeable differences in density or compactness. Bone-derived samples, ASC-B (Figure 3A-3) and PSC-B (Figure 3A-4), display relatively denser networks compared to skin-derived samples, suggesting that the mineral matrix in bone tissue contributes to a higher degree of intermolecular cross-linking.

At medium magnifications (×2000; Figure 3B-1,B-2,B-3,B-4), the arrangement and details of the fibres become more evident. ASC-S (Figure 3B-1) and PSC-S (Figure 3B-2) show similarly well-defined fibrous structures with no significant differences in fibre density or pore size. In contrast, ASC-B (Figure 3B-3) and PSC-B (Figure 3B-4) exhibit denser networks, with PSC-B showing more compact and organised fibre bundles. This could be attributed to pepsin digestion altering the molecular structure and enhancing cross-linking [35].

At higher magnifications (×5000; Figure 3C-1, 3C-2, 3C-3, 3C-4), the microscopic details of the fibres are further revealed. ASC-S (Figure 3C-1) and PSC-S (Figure 3C-2) display relatively uniform fibrous structures, but no significant differences in fibre tightness or pore size are observed. Bone-derived samples, ASC-B (Figure 3C-3) and PSC-B (Figure 3C-4), show more compact and tightly arranged fibres, with PSC-B exhibiting the most organised bundles and smallest pores. This further supports the notion that bone-derived samples have higher cross-linking density, and pepsin digestion influences the microstructure.

Bone-derived collagen (ASC-B and PSC-B) demonstrates higher fibre density and more compact networks compared to skin-derived collagen (ASC-S and PSC-S), reflecting intrinsic differences in tissue composition. Pepsin-treated samples (PSC-S and PSC-B) exhibit particularly dense and compact structures, especially in bone-derived collagen, suggesting that enzymatic treatment modifies the microstructure of collagen fibres.

The fibrous and interconnected porous network observed in these collagen samples indicates their potential for biomedical applications, such as promoting cell attachment, proliferation, and drug delivery. This aligns with previous studies that highlight collagen’s significance in tissue engineering and wound healing [36].

### 3.7. Thermal Stability Analysis

The DSC thermograms of acid-soluble collagen (ASC) and pepsin-soluble collagen (PSC) from eel skin and bone are shown in Figure 4. All samples exhibited two distinct endothermic peaks. The first peak is associated with the thermal denaturation temperature of collagen and the release of water bound to the molecules. The *T_max_*_1_ values for ASC-S, ASC-B, PSC-S, and PSC-B were 74.34 °C, 81.39 °C, 79.86 °C, and 74.23 °C, respectively, with corresponding ΔH1 values of 9.88 J/g, 21.61 J/g, 3.14 J/g, and 23.11 J/g. These results are similar to those reported for northern pike collagen (ASC: 79.3 °C, PSC: 80.1 °C) [37] and silver carp collagen (ASC: 77 °C, PSC: 81 °C) [24]. The second endothermic peak (*T_max_*_2_) is related to the structural changes and degradation of the collagen cross-links. Our findings indicate that the *T_max_*_2_ values for bone collagen (148.30 °C and 172.47 °C) are slightly higher than those for skin collagen (90.01 °C and 108.73 °C), suggesting that collagen derived from bone exhibits greater heat resistance and structural stability than collagen derived from skin.

### 3.8. UV Absorption Analysis

**Figure 5:**
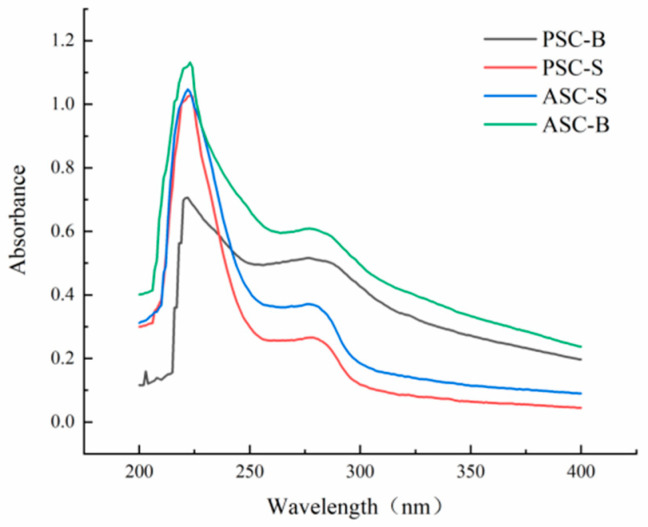
UV spectra of ASC-S, PSC-S, ASC-B, and PSC-B.

Figure 5 displays the UV absorption spectra of collagen extracted from eel skin and bone. Collagen molecules contain chromophoric groups such as –COOH, –CONH_2_, and –C=O, which exhibit strong UV absorption. The UV absorption spectrum is the result of the combined contributions of these chromophoric groups, making it a vital indicator for determining collagen type. The characteristic absorption peak of the collagen triple-helix structure typically appears in the wavelength range of 210–240 nm [38]. In this study, UV absorption spectra showed that both PSC and ASC had significant absorption peaks in this range, consistent with findings from sturgeon fish skin collagen [39], marine eel-fish (*Evenchelys macrura*) skin collagen [38], and balloon fish (*Diodon holocanthus*) skin collagen [40]. These results confirm the extracted collagen’s type I characteristics.

Additionally, due to the small amounts of tyrosine and phenylalanine present in collagen, a secondary absorption peak was observed at 280 nm. Among the samples, ASC-B demonstrated the most prominent peak at 280 nm. The absence of additional peaks beyond those near 223 nm and 280 nm indicates that the extracted collagen is of high purity with minimal contamination by other proteins. This is consistent with the FTIR and SEM results, indicating that ASCs and PSCs retain a high level of structural integrity throughout the extraction process.

The absorbance of acid-treated collagen (ASC) was notably higher than that of pepsin-treated collagen (PSC), as seen in Figure 5. This suggests that enzymatic treatment reduces collagen’s UV absorption capacity, likely due to partial structural disruption caused by enzymatic hydrolysis. Furthermore, among the samples, ASC-B exhibited the highest absorbance, indicating that acid treatment retained a more complete triple-helix structure compared to enzymatic treatment. These findings are consistent with the structural integrity observed in FTIR spectra and SEM images, which further confirm the higher purity and structural preservation of collagen treated with acid.

## 4. Discussion

This study comprehensively compared the biochemical, structural, and thermal properties of ASC and PSC derived from eel skin and bone. The results revealed distinct differences between skin- and bone-derived collagens, as well as the effects of extraction methods, providing valuable insights into the functional characteristics of these materials.

The difference in collagen yields between ASC and PSC reflect the influence of extraction methods on collagen recovery. The higher yields of PSC from both skin and bone align with the enhanced solubility imparted by pepsin, which cleaves non-helical telopeptides and facilitates the release of collagen from tightly cross-linked matrices. This trend is consistent with findings in loach skin [23] and silver carp skin [24], highlighting the efficiency of enzymatic extraction for tissues with high-density cross-links. However, the lower yields of ASC-B compared to ASC-S suggest that the cross-linking in bone tissue poses additional challenges for acid extraction, which primarily targets native triple-helical structures. This emphasises the importance of selecting appropriate extraction techniques to optimise collagen recovery based on tissue type.

The structural differences between ASC and PSC were confirmed by SDS-PAGE and FTIR analyses. While both methods verified the type I collagen composition of all samples, the additional low molecular weight bands in PSC samples reflect partial hydrolysis during enzymatic extraction. This hydrolysis, though potentially beneficial for applications requiring smaller peptides, may reduce the integrity of collagen’s triple-helical structure, as indicated by shifts in FTIR amide A and amide I bands. These observations suggest a trade-off between yield and structural preservation, with enzymatic extraction offering higher solubility but potentially altering collagen’s native structure. Previous studies on marine collagen have similarly reported structural disruptions in PSC compared to ASC [22,26,38,41].

Thermal stability analysis revealed that bone-derived collagen exhibits superior thermal resistance compared to skin-derived collagen, as evidenced by higher *T_max1_* and Δ*H_1_* values. This enhanced stability can be attributed to the dense fibrillar network and a higher degree of intermolecular cross-linking in bone collagen, as observed in SEM images. Interestingly, despite its lower *T_max1_*, skin collagen retained significant structural integrity, which may reflect differences in amino acid composition. The higher content of proline and hydroxyproline in bone collagen contributes to its stability, a characteristic noted in other fish species [28,29]. These results suggest that bone collagen may be more suitable for applications requiring structural robustness, such as tissue engineering, while skin collagen’s lower stability could make it advantageous for food and cosmetic applications.

The UV absorption spectra further highlight the purity and structural characteristics of the extracted collagens. The prominent absorption peaks at 223 nm confirm the presence of the collagen triple-helix structure, consistent with findings in balloon fish [40]. The peak at 280 nm, particularly pronounced in ASC-B, suggests higher aromatic amino acid content in bone collagen, potentially contributing to antioxidant activity. The absence of additional peaks indicates minimal contamination by non-collagenous proteins, underscoring the effectiveness of both acid and enzymatic extractions in yielding high-purity collagen.

The interplay between structural and functional properties observed across experiments highlights the influence of tissue origin and extraction method on collagen characteristics. For example, the denser microstructure and higher thermal stability of bone collagen correlate with its higher degree of cross-linking and distinct amino acid composition. In contrast, the relatively looser fibrillar arrangement and lower thermal stability of skin collagen reflect its adaptation for flexibility and ease of processing. These complementary properties suggest distinct yet overlapping application potentials for skin and bone collagen, ranging from food and cosmetics to biomedical uses.

Based on the findings of this study, bone-derived collagen (PSC-B) demonstrates higher thermal stability and a denser network structure, while skin-derived collagen exhibits more fibrous and porous characteristics. These differences provide valuable insights into the potential applications of collagen derived from different sources of eel. For instance, bone collagen, with its superior stability, is particularly suitable for applications requiring mechanical strength and thermal resistance, such as wound healing scaffolds and drug delivery systems. In contrast, skin collagen, characterised by its lower stability and ease of processing, may be more appropriate for functional food products and skincare formulations. These results contribute to the growing interest in utilising marine collagen as a sustainable alternative to mammalian collagen, particularly in addressing challenges related to zoonotic diseases and religious restrictions.

Future research should focus on exploring the bioactivity of collagen peptides derived from eel skin and bone, particularly their antioxidant and ACE inhibitory properties. Investigating the mechanical and biocompatibility properties of collagen-based materials in vivo would provide further validation for biomedical applications. Additionally, optimising extraction protocols to balance yield, purity, and structural preservation could enhance the scalability and economic feasibility of eel collagen production. Comparative studies across different marine species could also elucidate the relationship between tissue origin, collagen properties, and functional performance, further expanding the scope of marine collagen applications.

## Figures and Tables

**Table 1:** The comparison of proximate content of eel skin and bone.

Position	Moisture Mass Fraction (%)	Crude Protein Mass Fraction (%)	Crude Fat Mass Fraction (%)	Ash Mass Fraction (%)
Eel skin	75.54 ± 0.54	19.00 ± 0.46	1.40 ± 0.08	0.58 ± 0.03
Eel bone	46.25 ± 0.23	20.86 ± 0.07	25.92 ± 0.32	6.21 ± 0.24

**Table 2:** Amino acid composition of eel skin and bone collagen.

Amino Acid Category	The Proportion of Amino Acids (% of Total Amino Acids)			
	ASC-S	PSC-S	ASC-B	PSC-B
Asp	5.86	5.66	6.25	5.41
Thr	3.33	3.22	2.53	2.21
Ser	4.72	4.63	3.13	2.82
Glu	10.88	9.94	9.22	8.76
Gly	22.67	19.86	19.94	16.66
Ala	11.14	10.24	10.74	7.77
Cys	ND	ND	0.24	0.11
Val	1.89	2.15	2.45	3.13
Met	0.50	0.67	3.03	3.59
Ile	2.00	1.29	3.47	2.44
Leu	2.52	2.78	3.18	3.29
Tyr	0.31	0.43	0.34	0.39
Phe	2.11	1.87	2.25	2.13
His	1.32	0.82	1.28	1.15
Lys	3.97	3.42	3.67	3.04
Arg	6.99	8.34	8.77	14.19
Pro	14.89	16.91	7.23	7.71
Hyp	6.41	7.77	12.29	15.18
Essential amino acid	16.32	15.40	20.58	19.83
Hydrophobic amino acid	35.05	35.91	32.35	30.06
Aromatic amino acid	2.42	2.30	2.59	2.52

Note: ND, not determined. ASC-S, acid-soluble collagens from skin; PSC-S, pepsin-soluble collagens from skin; ASC-B, acid-soluble collagens from bone; PSC-B, pepsin-soluble collagens from bone.

## Data Availability

The original contributions presented in this study are included in the article. Further inquiries can be directed to the first author.
